# Aromatic L‐Amino Acid Decarboxylase Gene Therapy Enhances Levodopa Response in Parkinson's Disease

**DOI:** 10.1002/mds.27993

**Published:** 2020-03-09

**Authors:** John G. Nutt, Carolin Curtze, Amie Hiller, Shannon Anderson, Paul S. Larson, Amber D. Van Laar, R. Mark Richardson, Marin E. Thompson, Alexander Sedkov, Mika Leinonen, Bernard Ravina, Krystof S. Bankiewicz, Chadwick W. Christine

**Affiliations:** ^1^ Department of Neurology Oregon Health & Science University Portland Oregon USA; ^2^ Department of Biomechanics University of Nebraska at Omaha Omaha Nebraska USA; ^3^ Department of Neurological Surgery University of California San Francisco San Francisco California USA; ^4^ Department of Neurology University of Pittsburgh Pittsburgh Pennsylvania USA; ^5^ Department of Neurological Surgery University of Pittsburgh Pittsburgh Pennsylvania USA; ^6^ Department of Neurosurgery Massachusetts General Hospital Boston Massachusetts USA; ^7^ Voyager Therapeutics, Inc. Cambridge Massachusetts USA; ^8^ Clinical Data Science GmbH Basel Switzerland; ^9^ Department of Neurology University of California San Francisco San Francisco California USA; ^10^ Department of Neurological Surgery The Ohio State University Columbus Ohio USA

**Keywords:** AADC, gene therapy, levodopa, Parkinson's disease, VY‐AADC01

## Abstract

**Background:**

As Parkinson's disease progresses, levodopa treatment loses efficacy, partly through the loss of the endogenous dopamine‐synthesizing enzyme L‐amino acid decarboxylase (AADC). In the phase I PD‐1101 study, putaminal administration of VY‐AADC01, an investigational adeno‐associated virus serotype‐2 vector for delivery of the AADC gene in patients with advanced Parkinson's disease, was well tolerated, improved motor function, and reduced antiparkinsonian medication requirements.

**Objectives:**

This substudy aimed to determine whether the timing and magnitude of motor response to intravenous levodopa changed in PD‐1101 patients after VY‐AADC01 administration.

**Methods:**

Participants received 2‐hour threshold (0.6 mg/kg/h) and suprathreshold (1.2 mg/kg/h) levodopa infusions on each of 2 days, both before and approximately 6 months after VY‐AADC01. Infusion order was randomized and double blinded. Unified Parkinson's Disease Rating Scale motor scores, finger‐tapping speeds, and dyskinesia rating scores were assessed every 30 minutes for 1 hour before and ≥3 hours after start of levodopa infusion.

**Results:**

Of 15 PD‐1101 patients, 13 participated in the substudy. Unified Parkinson's Disease Rating Scale motor score area under the curve responses to threshold and suprathreshold levodopa infusions increased by 168% and 67%, respectively, after VY‐AADC01; finger‐tapping speeds improved by 162% and 113%, and dyskinesia scores increased by 208% and 72%, respectively, after VY‐AADC01. Adverse events (mild/moderate severity) were reported in 5 participants during levodopa infusions pre–VY‐AADC01 and 2 participants post–VY‐AADC01 administration.

**Conclusions:**

VY‐AADC01 improved motor responses to intravenous levodopa given under controlled conditions. These data and findings from the parent study support further clinical development of AADC gene therapy for people with Parkinson's disease. © 2020 The Authors. *Movement Disorders* published by Wiley Periodicals, Inc. on behalf of International Parkinson and Movement Disorder Society.

Parkinson's disease (PD) is characterized by the progressive loss of substantia nigra dopaminergic neurons that project to the striatum, with greater initial loss of projections to the posterior putamen.^1^, ^2^, ^3^, ^4^ Levodopa, the immediate precursor of dopamine, is the most effective treatment for the motor symptoms of PD.^5^, ^6^ Synthesis of dopamine from levodopa in nigrostriatal neurons is mediated by the enzyme L‐amino acid decarboxylase (AADC).^7^, ^8^ As disease progresses over time and AADC levels fall,^9^, ^10^ previously sufficient doses of levodopa lose efficacy; ultimately, patients experience a less robust and predictable levodopa response characterized by motor fluctuations.^6^, ^9^, ^11^, ^12^ Consequently, patients may require higher and additional doses of levodopa and adjunct therapies to maintain motor function. Unfortunately, higher doses of dopaminergic therapies for PD are often associated with dose‐limiting motor (such as dyskinesias)^13^, ^14^, ^15^, ^16^, ^17^ and nonmotor (gastrointestinal symptoms, neuropsychiatric symptoms, impulse control disorders, and hallucinations) side effects.^13^, ^14^, ^16^, ^17^, ^18^

To address the loss of dopamine synthesis from orally administered levodopa, the replacement of AADC enzymatic activity through adeno‐associated viral vector serotype‐2 (AAV2)–mediated gene therapy has been investigated in preclinical models and clinical trials. Direct infusion of AAV2s containing complementary DNA encoding the human AADC enzyme (AAV2‐hAADC) to the striatum of nonhuman primates (NHPs) with 1‐methyl‐4‐phenyl‐1,2,3,6‐tetrahydropyridine (MPTP)‐induced parkinsonism durably increased AADC activity in the striatum and led to improved clinical rating scores in response to low doses of levodopa that were ineffective in sham gene therapy–treated parkinsonian NHPs.^19^, ^20^, ^21^ In 2 initial clinical trials, conventional “blind” stereotactic administration of AAV2‐hAADC to the putamen of patients with PD was well tolerated and led to increased enzyme activity, as determined by 6‐[^18^F]fluoro‐L‐meta‐tyrosine positron emission tomography, that persisted for 4 years after surgery.^22^, ^23^, ^24^, ^25^ However, the clinical benefit observed in the patients enrolled in these trials was limited and not durable, possibly because of low vector infusion volumes and inadequate putaminal coverage.^22^, ^23^, ^25^

PD‐1101 is an open‐label, phase I, dose‐escalation trial of AADC gene therapy in patients with moderately advanced PD utilizing intraoperative magnetic resonance imaging–guided putaminal administration of VY‐AADC01, an AAV2 vector for the delivery of the gene encoding the AADC enzyme. In this trial, VY‐AADC01 was well tolerated and led to increased AADC enzyme activity (measured by ^18^F‐fluoro‐L‐dihydroxyphenylalanine positron emission tomography). Antiparkinsonian medications requirements decreased, patient‐reported *on* time without troublesome dyskinesia increased, Unified Parkinson's Disease Rating Scale (UPDRS) III scores decreased, and quality of life improved.^26^

Intravenous (IV) levodopa challenge has been used in previous studies to independently and objectively evaluate the effect of potential therapies on motor responses to levodopa in people with PD.^27^, ^28^ These studies quantified responses to 2 different doses of levodopa in a randomized and blinded fashion to allow for an objective assessment of clinical effects of the evaluated therapies. Here we report the outcomes of a substudy of PD‐1101 in which the responsiveness to 2 different doses of IV levodopa was examined prior to and following VY‐AADC01 administration using a blinded and randomized study design. Exploiting the ability of AADC to convert levodopa to dopamine, this gene therapy approach was hypothesized to increase putaminal dopamine availability that would consequently enhance the duration and/or magnitude of responses to fixed doses of levodopa, measured by motor function tests. Full methods and interim safety and efficacy outcomes of the PD‐1101 trial have been reported previously.^26^

## Methods

### Participants

This substudy of PD‐1101 was conducted at Oregon Health & Science University (OHSU). Patients enrolled in PD‐1101 who were able to travel to OHSU participated in this substudy. Patients eligible for inclusion in PD‐1101 were aged 40 to 70 years and had PD with medically refractory motor fluctuations, a history of responsiveness to dopaminergic therapy, and disease duration ≥5 years; further details on inclusion and exclusion criteria can be found at https://clinicaltrials.gov (NCT01973543).

### VY‐AADC01 Dosing and Surgical Administration

Details on preparation and surgical administration of VY‐AADC01 have been described previously.^26^ Briefly, patients received bilateral intraoperative magnetic resonance imaging–guided administration of the gene therapy to the putamen using a frontal approach and ≥2 trajectories per hemisphere. There were 3 dose cohorts: cohort 1, total dose ≤7.5 × 10^11^ vector genomes in an infusion volume of ≤450 μL per putamen; cohort 2, ≤1.5 × 10^12^ vector genomes in ≤900 μL; cohort 3, ≤4.7 × 10^12^ vector genomes in ≤900 μL.

### IV Levodopa Assessment

The protocol for IV levodopa assessment was approved by the OHSU institutional review board, and all participants provided written informed consent. Participants underwent IV levodopa assessment prior to and ≈6 months after administration of VY‐AADC01. They were admitted to the hospital on the day prior to first levodopa administration and underwent baseline examination. All antiparkinsonian medications (including immediate and controlled‐release formulations) were withheld from 10:00 pm the night prior to assessment until the completion of assessment on the following day. Participants received levodopa infusions at 2 different dose levels over 2 days, with 1 infusion per day. Infusions were prepared as 0.6 mg/mL (threshold concentration) and 1.2 mg/mL (suprathreshold concentration) solutions and delivered intravenously at 1 mL/kg/h for 2 hours each day. The order in which the 2 levodopa doses were delivered was randomized for each participant by the research pharmacy, and investigators and participants were blinded to dosing. This paradigm was based on earlier studies, with increased doses compared with those used previously (0.5 mg/mL and 1.0 mg/mL, respectively).^27^, ^28^ The threshold dose was expected to represent a threshold for clinical responsiveness, such that participants might receive a brief clinical benefit or none at all, whereas the suprathreshold dose was predicted to almost certainly elicit clinical responses. The threshold and suprathreshold doses of levodopa closely resembled those used in the aforementioned primate studies, in which MPTP‐lesioned NHPs that received AAV2‐hAADC responded to low‐dose and high‐dose levodopa infusions, whereas MPTP‐lesioned animals that received sham gene therapy only responded to high‐dose levodopa.^20^ We did not include a placebo dose because admissions to the clinical research unit were limited to 2 days given the taxing nature of the protocol for participants; based on the rationale noted previously, we felt the threshold and suprathreshold doses of greater utility. In addition, given that the informed consent forms would give participants prior knowledge that 1 dose would be placebo, it was considered likely that participants would identify the placebo dose within 15 to 60 minutes of infusion initiation, confounding assessment of clinical responses. Oral carbidopa (25 mg) was administered 1 hour prior to, 1 hour after, and 3 hours after initiation of levodopa infusion to match the 2‐hour treatment intervals often employed in clinical use for patients with moderately advanced PD.

Starting 1 hour prior to initiation of levodopa infusion, assessment of UPDRS III scores, finger‐tapping speed, dyskinesia scores, and electrocardiogram rhythm strips were done every 30 minutes until after the infusion was completed and clinical measures returned to the preinfusion baseline values for 2 consecutive assessments, with a minimum of 3 hours of monitoring after initiation of infusion. Blood sampling for plasma levodopa concentrations was done immediately prior to infusion, then at 30‐minute intervals for 3 hours, then at 1‐hour intervals until the end of clinical monitoring.

Primary outcome measures were UPDRS III (motor section of the UPDRS) score^29^ and finger‐tapping speed, the number of times the patient could, in 1 minute, alternately tap 2 counters placed 20 cm apart using the index finger of their more affected arm.^30^ Finger‐tapping speed is a validated measure of bradykinesia in PD.^31^, ^32^ Secondary outcome measures included dyskinesia and plasma levodopa levels. Dyskinesia was assessed using a modified version of the previously developed dyskinesia rating scale,^30^ with a score of 0 (absent) to 4 (incapacitating) for 7 body parts (each limb, face, neck, and trunk) for a maximum possible score of 28. Safety was evaluated by assessment of incidence of electrocardiogram changes (to detect arrhythmias), adverse events (AEs), and vital sign abnormalities.

### Statistical Analysis

UPDRS III scores, finger‐tapping speeds, and dyskinesia scores are presented as change from baseline over time (time‐action curves). Because there was some improvement in *off*‐medication scores after gene therapy, baselines for all time‐action curves were the mean of the −1 hour, −0.5 hour, and 0 hour time points from both pre–VY‐AADC01 days (ie, the mean of the 6 preinfusion, pre–VY‐AADC01 values). After the start of levodopa infusion, UPDRS III scores greater than baseline and finger‐tapping speeds less than baseline were truncated to 0. As clinical responses may worsen to below baseline after levodopa wearing‐off^33^ and the duration of response to infused levodopa varied among participants, including scores below baseline as a result of this *off* phenomenon could have obscured the tail end of the overall mean response. To avoid this, we truncated such scores, in alignment with previous levodopa challenge studies.^27^ Area under the curve (AUC) values were determined from time‐action curves.

Temporal aspects of IV levodopa response were measured in 3 ways: change from baseline at 30 minutes after start of infusion (for UPDRS III scores, finger‐tapping speeds, and dyskinesia rating scores), linearly extrapolated time to 30% reduction in score (latency to onset, UPDRS III only), and duration of ≥30% reduction in score (UPDRS III only). For all measures of timing, baseline was the value at the 0 hour time point (initiation of levodopa infusion) on the day of assessment. A 30% change in UPDRS III as a threshold for response has been employed in a previous trial of a PD medication.^34^

Peak responses were the minimum absolute values for UPDRS III scores and the maximum absolute values for finger‐tapping speed and dyskinesia rating scale scores. Data are reported as mean ± standard error of the mean.

## Results

### Participants

Of the 15 patients enrolled in PD‐1101, 13 participated in this substudy: all 5 patients from cohort 1, all 5 patients from cohort 2, and 3 of 5 patients from cohort 3. A total of 2 patients from cohort 3 were unable to travel to OHSU. Demographics and baseline characteristics for participants in the substudy were generally well balanced between cohorts, although UPDRS III scores on medication were lower in cohort 1 than the other 2 cohorts, and Unified Dyskinesia Rating Scale scores were higher in cohort 3 than the other 2 cohorts (Supplementary Table S1). One participant in cohort 1 did not achieve *on* status in response to threshold‐dose IV levodopa at the pre–VY‐AADC01 visit and refused several assessments. Last observation carried forward values were imputed for missing data values from this participant.

### Clinical Outcomes

**Figure 1:**
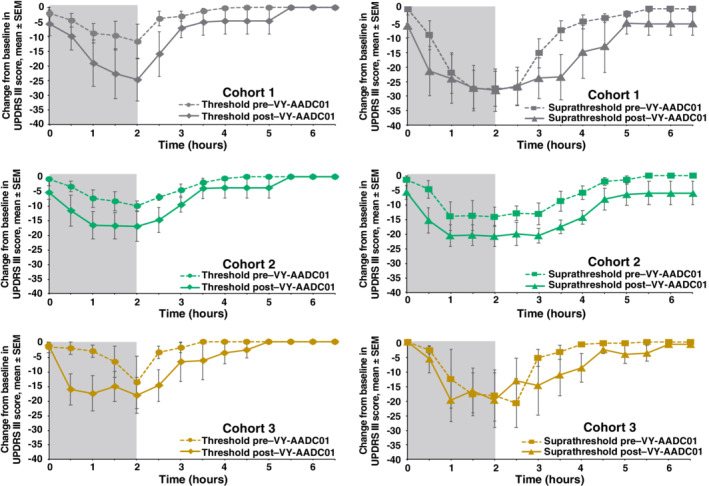
Change from baseline in UPDRS III scores over time during and following threshold and suprathreshold infusions of intravenous levodopa pre– and post–VY‐AADC01 administration. A reduction in UPDRS III scores represents improvement. Gray shaded area shows actual time of infusion. Figure is a modified reproduction from reference 26, used with permission under the author reuse policy of the publisher. SEM, standard error of the mean; UPDRS, Unified Parkinson's Disease Rating Scale.

**Figure 2:**
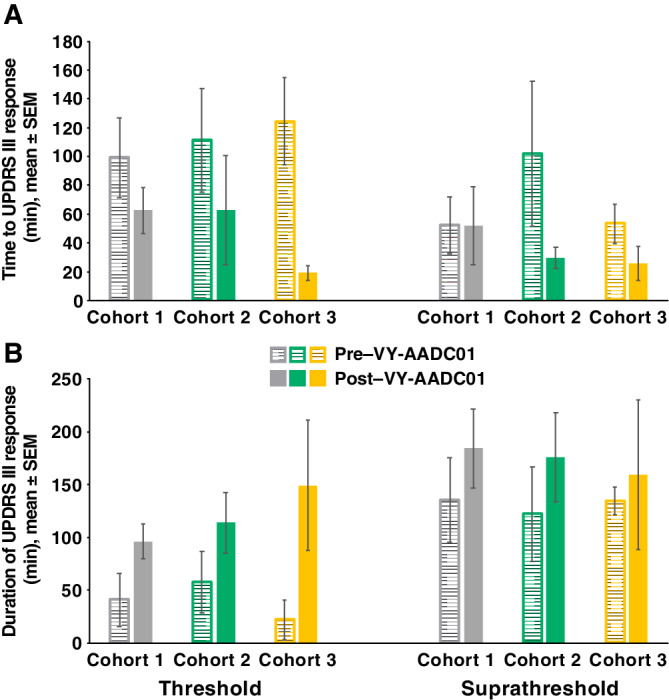
Time to 30% change in UPDRS III scores (**A**) and duration of ≥30% UPDRS response (**B**) after threshold and suprathreshold IV levodopa infusion pre– and post–VY‐AADC01 administration. IV, intravenous; SEM, standard error of the mean; UPDRS, Unified Parkinson's Disease Rating Scale.

The effects of VY‐AADC01 on the UPDRS III scores were most prominent with the threshold (lower dose) levodopa infusions. Magnitude and duration of responses to threshold levodopa were increased after gene therapy in all cohorts, as shown by time‐action curves (Fig. 1). The 168% increase in mean AUC and marked improvement in peak response (Supplementary Table S2) supported the time‐action curve findings. UPDRS III responses were more rapid in onset and more persistent after gene therapy: time to 30% reduction (latency of onset) was shortened and duration of ≥30% response was prolonged in all cohorts (Fig. 2). Change from baseline at 30 minutes was also increased, further supporting a more rapid response. For suprathreshold levodopa infusions, time‐action curves showed that the gene therapy increased the duration of UPDRS III responses in all cohorts but increased the magnitude only in cohort 2. AUC increased by 67%, with minimal improvement in peak response. Latency of onset was shortened in cohorts 2 and 3, and there were modest increases in duration of ≥30% response in each cohort. Change from baseline at 30 minutes also increased after VY‐AADC01.

**Figure 3:**
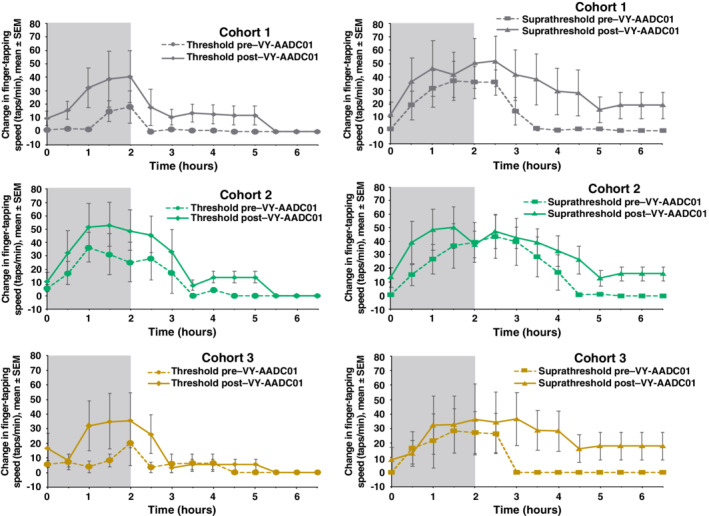
Change from baseline in finger‐tapping speed over time during and following threshold and suprathreshold infusions of intravenous levodopa pre– and post–VY‐AADC01 administration. Increase in finger‐tapping speed represents improvement. Gray shaded area shows actual time of infusion. SEM, standard error of the mean.

Finger‐tapping speeds, an independent measure of bradykinesia, also improved after AADC gene delivery. Time‐action curves for finger‐tapping in the threshold and suprathreshold condition largely mirrored those for UPDRS III scores (Fig. 3). Large increases in AUC were observed after VY‐AADC01 for both levodopa doses (threshold, 162%; suprathreshold, 113%), whereas increases in peak response and change from baseline at 30 minutes were modest (Supplementary Table S2).

**Figure 4:**
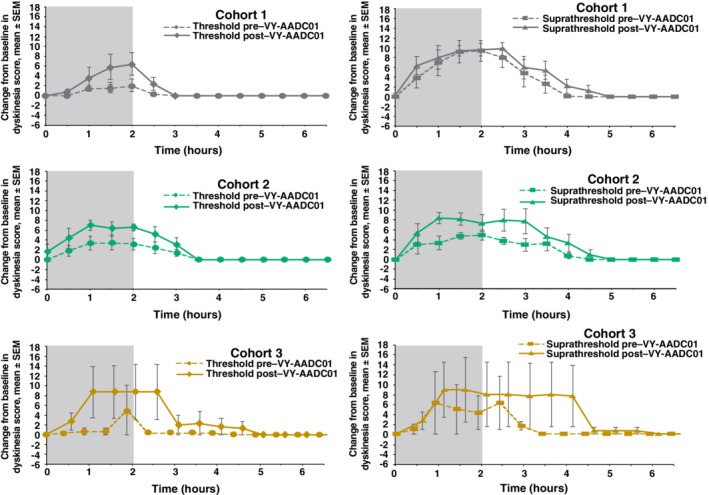
Change from baseline in dyskinesia rating scale scores during and following threshold and suprathreshold infusions of intravenous levodopa pre– and post–VY‐AADC01 administration. Increase in dyskinesia rating scale score indicates worsened dyskinesia. Gray shaded area shows actual time of infusion. SEM, standard error of the mean.

Not surprisingly, dyskinesia scores with levodopa infusion were higher after gene therapy (Fig. 4). The duration of dyskinesia was longer post–VY‐AADC01 administration in all cohorts in the suprathreshold condition. Mean overall AUC for dyskinesia increased by 208% and 72% in the threshold and suprathreshold levodopa conditions, respectively. There were also small increases in dyskinesia peak response to levodopa infusion after gene therapy as well as in change from baseline at 30 minutes (Supplementary Table S2).

**Figure 5:**
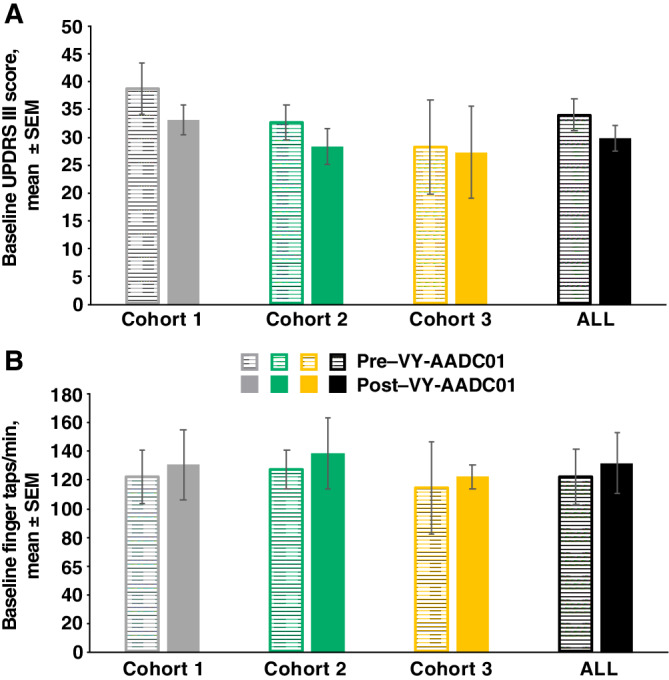
UPDRS III scores (**A**) and finger‐tapping speeds (**B**) at baseline of intravenous levodopa challenge pre– and post–VY‐AADC01 administration. Baseline score for each participant is the mean of baseline scores from threshold and suprathreshold infusion days. A reduction in UPDRS III score represents improvement. Increase in finger‐tapping speed represents improvement. SEM, standard error of the mean; UPDRS, Unified Parkinson's Disease Rating Scale.

After VY‐AADC01 administration, there were trends toward improvement in off‐medication UPDRS III scores and finger‐tapping speeds at baseline (ie, when the patients had been without levodopa and other antiparkinsonian medications overnight; Fig. 5; also note apparent baseline shifts in Figs. 1 and 3).

Mean plasma levodopa concentration profiles during and after the levodopa infusions were similar before and after VY‐AADC01 treatment (Supplementary Fig. S1).

### Safety

AEs were reported in 5 participants during levodopa challenges; 5 had AEs during the pre–VY‐AADC01 levodopa challenge, and 2 also had AEs during the post–VY‐AADC01 levodopa infusions. All AEs were mild or moderate in severity (Supplementary Table S3). No arrhythmias were detected with electrocardiogram monitoring. Safety findings from the parent PD‐1101 study have been reported previously.^26^

## Discussion

VY‐AADC01 gene therapy was designed to increase conversion of levodopa to dopamine in the putamen of patients with PD. The changes in ^18^F‐fluoro‐L‐dihydroxyphenylalanine positron emission tomography signal observed after gene therapy administration in the parent study confirmed increased AADC enzymatic activity.^26^ The increased AADC activity was associated with a consistent increase in clinical response to IV levodopa in this substudy. Enhanced responses to levodopa infusion were not the result of increased peripheral levodopa, as there were no differences in plasma levodopa concentrations during IV challenge before and after VY‐AADC01 administration (Supplementary Fig. S1). This gene therapy particularly augmented the response to low‐dose levodopa infusions, suggesting that efficacious dopamine levels may be reached at lower levodopa doses. This observation is consistent with previous studies in NHPs with MPTP‐induced parkinsonism, in which AAV2‐hAADC gene therapy enhanced clinical responses to low doses of levodopa that were ineffective in parkinsonian animals that did not receive AAV2‐hAADC.^20^

The changes in UPDRS III scores and finger‐tapping speeds, particularly in the suprathreshold levodopa condition, were driven primarily by decreased latency to effect onset and increased effect duration, with a minor contribution from increases in peak responses. These findings align with previous evidence that increasing plasma levodopa concentrations above minimally effective levels enhances the duration of improvements in finger‐tapping and walking speed, but not the magnitude of such responses.^35^ In an evaluation of levodopa therapeutic windows, the magnitude of motor function responses to IV levodopa plateaued at approximately 150% of threshold dose in patients with advanced PD, whereas the duration of responses continued to increase at doses 300% above threshold.^36^ A possible explanation for this disconnect between magnitude and duration of effect may be that dopamine synthesis sufficient to saturate dopamine receptors is reached at some threshold levodopa dose, and levodopa in excess of this threshold only prolongs the duration of response. After VY‐AADC01 gene therapy, this threshold level of putaminal dopamine may be reached with lower levodopa doses because of the enhanced local conversion of levodopa to dopamine.

Dyskinesia and antiparkinsonian benefits generally go hand in hand in people with PD who have a fluctuating response to levodopa. Therefore, an increase in dyskinesia that paralleled the increase in clinical response was not unexpected.^36^, ^37^, ^38^ In the parent study, dyskinesias did increase post–VY‐AADC01 administration as anticipated with the predicted mechanism of action; these dyskinesias proved to be transient and uniformly responsive to reductions in antiparkinsonian medications and ultimately were not worse than before gene therapy.^26^

An unanticipated finding in this substudy was that baseline UPDRS III scores and finger‐tapping speeds improved after VY‐AADC01 administration. This observation is consistent with unpublished findings in NHPs with MPTP‐induced parkinsonism in which clinical rating scores gradually improved after AADC gene therapy in the absence of levodopa treatment (K.S. Bankiewicz, personal communication, March 2019). The present findings are also in agreement with improvements in *off*‐medication UPDRS III scores observed in previous clinical studies of AAV2‐hAADC^22^, ^24^ and in the parent study.^26^ A possible explanation for this improvement in the *off*‐medication condition is increased synthesis of dopamine from endogenous levodopa. In pharmacokinetic/pharmacodynamic models of levodopa action in PD, the inclusion of low levels of endogenous levodopa production as a factor improved predictions of actual patient‐derived data.^39^

A limitation of this substudy is the potential for a placebo effect following gene therapy administration; placebo effects are particularly prominent after neurosurgical interventions in PD.^40^ In addition, PD‐1101 had small cohort sizes that were not powered for efficacy assessments or for between‐cohort comparisons. A lingering question is whether the administration of IV levodopa at a clinical research center translates to the clinical setting with oral levodopa and the use of other antiparkinsonian medications. The findings of this substudy predicted that antiparkinsonian medication needs would be decreased, consistent with the actual reductions in medication use by these patients reported in the parent study.

In conclusion, the IV levodopa paradigm provided a blinded and more objective examination of the effects of VY‐AADC01 gene therapy on responses to levodopa in PD. The results were consistent with the clinical responses in the patients and confirm and extend the findings in NHPs. The substudy provides further evidence that AADC gene therapy may provide meaningful benefits to people with PD and supports further clinical development of VY‐AADC01.

## Author Roles

(1) Research project: A. Conception, B. Organization, C. Execution; (2) Statistical Analysis: A. Design, B. Execution, C. Review and Critique; (3) Manuscript: A. Writing of the first draft, B. Review and Critique.

J.G.N.: 1A, 1B, 1C, 2A, 3A, 3B

C.C.: 2A, 2B, 3B

A.H.: 1B, 1C, 3B

S.A.: 1B, 1C, 3B

P.S.L.: 1A, 1B, 1C, 3B

A.D.V.L.: 1B, 1C, 3B

R.M.R.: 1B, 1C, 3B

M.E.T.: 1B, 1C, 3B

A.S.: 2A, 2B, 3B

M.L.: 2A, 2B, 3B

B.R.: 1A, 1B, 3B

K.S.B.: 1A, 1B, 3B

C.W.C.: 1A, 1B, 1C, 3B

## Financial disclosures of all authors (for the preceding 12 months)

J.N., A.D.V.L., R.M.R., and C.W.C. received grants from Voyager Therapeutics, Inc. P.S.L. received grants from Voyager Therapeutics and nonfinancial support from MRI Interventions, Inc. M.E.T. received grants and nonfinancial support from Voyager Therapeutics. A.S. is an employee of Voyager Therapeutics and owns stock in that company. M.L. has provided paid statistical consultation services for Voyager Therapeutics. B.R. is a former employee of Voyager Therapeutics. K.S.B. received grants and personal fees from Voyager Therapeutics. C.C., A.H., and S.A. have nothing to disclose.

## Supporting information


**Supplementary Table 1**. Demographics and baseline characteristics.
**Supplementary Table 2**. Analyses of area under the curve, peak response, and change from baseline at t = 30 min for UPDRS III score, finger‐tapping speed, and dyskinesia score in response to IV levodopa infusion pre– and post–VY‐AADC01 administration.
**Supplementary Table 3**. Adverse events during levodopa infusion.
**Supplementary Fig. 1**. Plasma concentrations of levodopa during and after IV levodopa infusion.
