# Supplementary material for: Aromatic L‐Amino Acid Decarboxylase Gene Therapy Enhances Levodopa Response in Parkinson's Disease
Source: Mov Disord. 2020 Mar 9;35(5):851–8. doi: 10.1002/mds.27993 (PMC7318280; doi:10.1002/mds.27993)
Supplement: Supplementary file 1 — Supplementary Table 1. Demographics and baseline characteristics. Supplementary Table 2. Analyses of area under the curve, peak response, and change from baseline at t = 30 min for UPDRS III score, finger‐tapping speed, and dyskinesia score in response to IV levodopa infusion pre– and post–VY‐AADC01 administration. Supplementary Table 3. Adverse events during levodopa infusion. Supplementary Fig. 1. Plasma concentrations of levodopa during and after IV levodopa infusion. [file MDS-35-851-s001.docx]

**SUPPLEMENTARY MATERIALS**

**Supplementary Table 1.** Demographics and baseline characteristics.

|  | **Cohort 1 ≤7.5×10^11^ vg N=5** | **Cohort 2 ≤1.5×10^12^ vg N=5** | **Cohort 3 ≤4.7×10^12^ vg N=3** | **Total N=13** |
| --- | --- | --- | --- | --- |
| Age, y | 57.4 (3.2) | 58.4 (3.9) | 58.7 (3.4) | 58.1 (1.9) |
| Male, n (%) | 4 (80) | 5 (100) | 2 (67) | 11 (85) |
| Duration of PD, y | 9.9 (2.1) | 10.1 (0.7) | 6.9 (2.3) | 9.3 (1.0) |
| UPDRS II off medication | 13.6 (0.9) | 16.0 (0.8) | 16.7 (4.7) | 15.2 (1.1) |
| UPDRS II on medication | 3.0 (1.3) | 3.6 (0.7) | 3.3 (2.4) | 3.3 (0.7) |
| UPDRS III off medication | 37.2 (2.6) | 35.8 (3.4) | 35.7 (5.8) | 36.3 (1.9) |
| UPDRS III on medication | 7.6 (2.3) | 17.0 (1.7) | 15.3 (2.3) | 13.0 (1.7) |
| Hauser diary OFF time, h^a^ | 4.9 (0.8) | 4.2 (0.6) | 4.9 (0.7) | 4.7 (0.4) |
| Hauser diary good ON time, h^a,b^ | 10.5 (1.0) | 10.7 (0.8) | 9.8 (0.5) | 10.4 (0.5) |
| Modified H&Y stage, n per stage | Stage 3: 5 Stage 4: 0 | Stage 3: 5 Stage 4: 0 | Stage 3: 2  Stage 4: 1 | Stage 3: 12  Stage 4: 1 |
| UDysRS total score^c^ | 19.2 (6.0) | 17.4 (5.6) | 27.7 (1.5) | 20.5 (3.2) |
| Levodopa equivalent dose | 1467.5 (275.0) | 1635.5 (307.4) | 1307.5 (101.3) | 1495.2 (153.4) |

Data are mean (standard error of the mean) unless specified.

^a^Hauser diary data normalized to a 16-hour day.

^b^Good ON time is defined as ON time without troublesome dyskinesia.

^c^UDysRS was assessed at PD-1101 baseline but not used to assess dyskinesia in this substudy.

H&Y, Hoehn and Yahr; PD, Parkinson’s disease; UDysRS, Unified Dyskinesia Rating Scale; UPDRS, Unified Parkinson’s Disease Rating Scale; vg, vector genomes.

**Supplementary Table 2.** Analyses of area under the curve, peak response, and change from baseline at t=30 min for UPDRS III score, finger-tapping speed, and dyskinesia score in response to IV levodopa infusion pre– and post–VY-AADC01 administration.

|  | **0.6 mg/kg/h levodopa infusion** | | **1.2 mg/kg/h levodopa infusion** | |
| --- | --- | --- | --- | --- |
|  | **Pre** | **Post** | **Pre** | **Post** |
| **Area under the curve^a^** | | | | |
| UPDRS III | -20.5 (4.7) | -55.0 (12.7) | -54.8 (8.4) | -91.5 (18.2) |
| Tapping (/min) | 47.7 (16.0) | 124.8 (34.6) | 96.9 (24.0) | 206.3 (43.7) |
| Dyskinesia | 5.0 (1.5) | 15.4 (4.3) | 16.7 (3.6) | 28.8 (5.0) |
| **Peak response^b^** | | | | |
| UPDRS III | 21.2 (2.7) | 12.8 (1.9) | 11.5 (2.1) | 9.1 (1.7) |
| Tapping (/min) | 152.2 (8.8) | 170.5 (10.7) | 163.1 (9.5) | 178.0 (9.8) |
| Dyskinesia | 4.0 (1.2) | 8.0 (1.4) | 8.0 (1.6) | 10.6 (1.4) |
| **Change from baseline at t=30 min^a^** | | | | |
| UPDRS III | -2.2 (1.3) | -8.8 (2.3) | -6.5 (2.6) | -12.0 (2.8) |
| Tapping (/min) | 4.2 (5.2) | 10.8 (5.5) | 18.9 (7.4) | 23.2 (6.0) |
| Dyskinesia | 0.8 (0.5) | 2.0 (0.9) | 2.8 (1.0) | 5.0 (1.1) |

Data are mean (standard error of the mean).

^a^Decrease in UPDRS III score represents improvement. Increase in finger-tapping speed represents improvement. Increase in dyskinesia score represents worsened dyskinesia.

^b^Peak responses represent mean of absolute minimum (best) UPDRS III scores, absolute maximum (best) finger-tapping speeds, and absolute maximum (worst) dyskinesia rating scores.

IV, intravenous; UPDRS, Unified Parkinson’s Disease Rating Scale.

**Supplementary Table 3.** Adverse events during levodopa infusion.

| **Participant with adverse event** | **Visit** | **Adverse event** | **Severity** |
| --- | --- | --- | --- |
| 1 | Pre–VY-AADC01 | Hot flash | Mild |
| 2 | Pre–VY-AADC01 | Headache | Mild |
|  | Post–VY-AADC01 | Tachycardia | Moderate |
|  |  | Headache | Moderate |
| 3 | Pre–VY-AADC01 | Hypotension | Moderate |
|  |  | Hypertension | Moderate |
|  | Post–VY-AADC01 | Sleepiness | Moderate |
| 4 | Pre–VY-AADC01 | Exacerbation of PD  OFF symptoms | Moderate |
|  |  | Anxiety | Moderate |
| 5 | Pre–VY-AADC01 | Adverse levodopa reaction^a^ | Moderate |

^a^Sleepiness, foot cramp, and palpitations, all of which arose 15 minutes after start of infusion and resolved after 30 minutes during the infusion without intervention; the participant reported that they commonly experienced these reactions to levodopa treatment.

PD, Parkinson’s disease.

**Supplementary Fig. 1.** Plasma concentrations of levodopa during and after IV levodopa infusion.

**0.6 mg/kg/h**


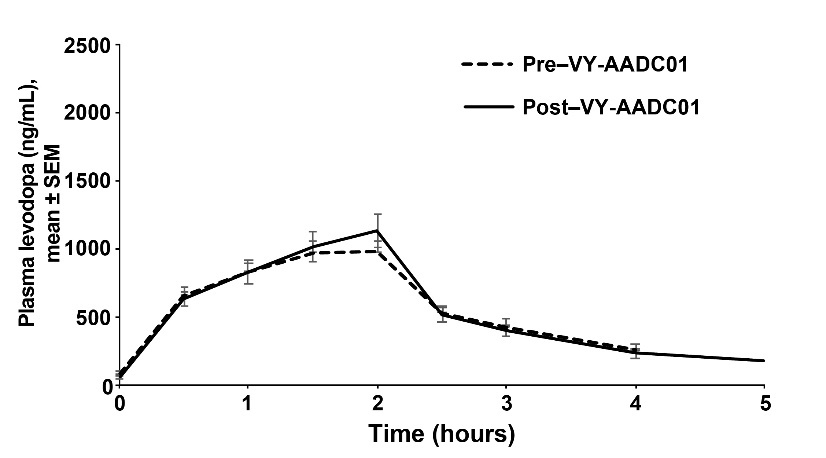


**1.2 mg/kg/h**


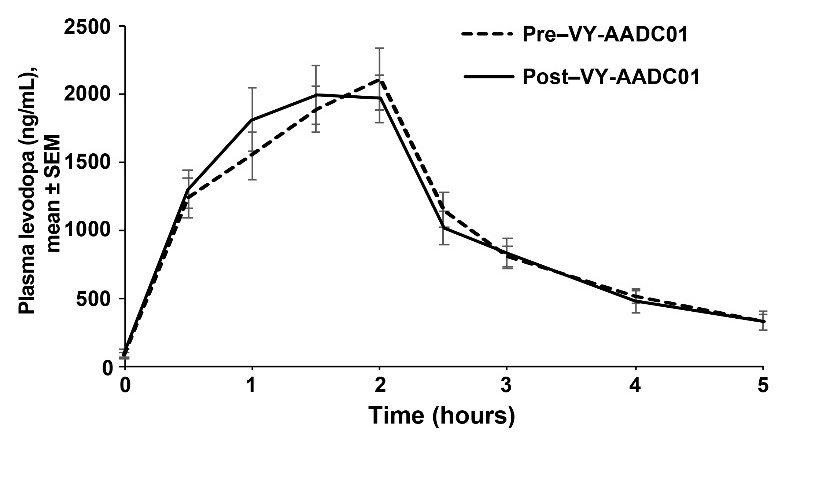


IV, intravenous, SEM, standard error of the mean.
